# 31547 Regulation and function of the i6A37 tRNA modification

**DOI:** 10.1017/cts.2021.407

**Published:** 2021-03-30

**Authors:** Joseph I Aubee, Kinlyn Williams, Alexandria Adigun, Olufolakemi Olusanya, Karl M Thompson

**Affiliations:** 1 Department of Microbiology, College of Medicine, Howard University; 2 Department of Biology, Claflin University; 3 Department of Biology, Howard University

## Abstract

ABSTRACT IMPACT: MiaA has a human homolog known as TRIT1. Mutations in TRIT1 have been associated with rare diseases such as MELAS and MERRF syndromes. These diseases are associated with mitochondrial disfunction.Understanding the mechanisms of bacterial sRNAs, and the miRNAs associated with these diseases could potentially afford the insight into effective cures. OBJECTIVES/GOALS: The aim is to investigate the regulation and function of tRNA isopentyladenine transferase enzyme in Escherichia coli. We aimed to execute screens for the identification of small RNA regulators of MiaA. The study will also investigate if i6A tRNA modification is necessary for the expression of major heat shock and mitochondrial proteins. METHODS/STUDY POPULATION: We constructed a chromosomal miaA-lacZ translational fusion driven by the arabinose responsive PBAD promoter and used it to screen against an Escherichia coli small RNA library. Using CsrB, one of our candidate sRNA regulators from our genetic screen, we measured the steady state levels of MiaA by Northern Blot in a PBAD-miaA2(P2HS)-lacZ translational fusion strain whereby pBR-pLac-csrB, pBR-pLac-csrA and the pBR-pLac vector are over-expressed, and under the control of an IPTG inducible promoter. Additionally, and in the same PBAD-miaA2(P2HS)-lacZ translational fusion strain background, we measured the steady state levels of MiaA in the wild type, csrA:zeo mutant strain, and csrA:zeo pBR-pLac-csrA complementation strain to determine if a combination of the pair would restore the wild-type genotype. RESULTS/ANTICIPATED RESULTS: Upon measuring the effect of small RNAs on miaA expression using quantitative b-galactosidase assays, we saw a 5-fold decrease in the expression of MiaA in the miaA-lacZ translational fusion containing sRNA CsrB, suggesting that this sRNA may play a role in the regulation of post-transcriptional expression of MiaA.From our northern blotting analysis, we observed a 6-fold decrease in MiaA expression in the absence of csrA, suggesting that csrA is essential for MiaA expression. DISCUSSION/SIGNIFICANCE OF FINDINGS: Identifying, mapping and characterizing how MiaA is regulated post-transcriptionally will give us an increased understanding in the maintenance and regulation of the normal function of E.coli to conserve homeostasis and translation fidelity.

